# Benzoylaconitine Alleviates Progression of Psoriasis via Suppressing STAT3 Phosphorylation in Keratinocytes

**DOI:** 10.3390/molecules28114473

**Published:** 2023-05-31

**Authors:** Yuanbo Li, Dandan Guo, Qianqian Wang, Aifang Li, Sugai Yin, Shuxuan Li, Yalan Li, Baiyan Wang, Tao Guo, Shuying Feng

**Affiliations:** 1 Medical College, Henan University of Chinese Medicine, Zhengzhou 450046, China; li_yuanbo1992@163.com (Y.L.); dandanguo163163@163.com (D.G.); wangqianhoney@126.com (Q.W.); liaifang-2020@hactcm.edu.cn (A.L.); yinsugai@163.com (S.Y.); lishuxuan37@163.com (S.L.); ly1001liyalan@163.com (Y.L.); baiyanw@hactcm.edu.cn (B.W.); 2 Key Laboratory of Human Functional Genomics of Jiangsu Province, Department of Neurobiology, School of Basic Medical Sciences, Nanjing Medical University, Nanjing 211166, China; 3 Department of Pharmacy, Henan University of Chinese Medicine, Zhengzhou 450046, China; gt010010@163.com

**Keywords:** Benzoylaconitine, psoriasis, HaCaT keratinocytes, inflammatory cytokine, STAT3 signaling

## Abstract

Psoriasis is a chronic and multifactorial skin disease which is caused by inflammatory infiltrates, keratinocyte hyperproliferation, and accumulation of immune cells. As part of the *Aconitum* species, Benzoylaconitine (BAC) shows potential antiviral, anti-tumor, and anti-inflammatory effects. In this study, we investigated the effects and mechanisms of BAC on tumor necrosis factor-alpha (TNF-α)/LPS-induced HaCaT keratinocytes in a imiquimod(IMQ)-induced mice model. The results showed that BAC could relieve the symptoms of psoriasis by inhibiting cell proliferation, the release of inflammatory factors, and the accumulation of Th17 cells, while no obvious effect on cell viability and safety was observed both in vitro and in vivo. Additionally, BAC can markedly inhibit the protein and mRNA levels of inflammatory cytokines in TNF-α/LPS-induced HaCaT keratinocytes by inhibiting the phosphorylation of STAT3. In brief, our data indicated that BAC could alleviate the progression of psoriasis and may be a potential therapeutic agent for treating psoriasis in clinical practice.

## 1. Introduction

Psoriasis is an auto-immune skin inflammation caused by genetic, immunological, or environmental factors, which affects 1–4% of people worldwide [1,2]. The pathophysiology of psoriasis includes erythematous plaque formation, epidermal hyperplasia, scaling, and impaired differentiation of keratinocytes [3,4,5]. Several factors participate in psoriasis, such as genetic susceptibility, pathogens, stress, drugs, etc. [6]. Moreover, most patients are also at risk for other diseases, including psoriasis arthritis, inflammatory bowel disease, cardiovascular disease, psychological status, and other comorbidities [7]. The current treatments for psoriasis mainly focus on local treatment, systemic treatment, and phototherapy [8]. Several studies have reported that the interleukin (IL) IL-17, IL-23, IL-22, and tumor necrosis factor-α (TNF-α) play a vital role in the epidermis of patients [9]. Furthermore, the accumulation of autoreactive skin T-cells and the activation of keratinocytes are key factors leading to a pro-inflammatory state [10,11]. Topical therapy, a conventional approach to treat psoriasis, is usually an incomplete lesion resolution [12]. Local treatment is not suitable for all patients with psoriasis for several reasons: low skin retention rate of drug preparations, poor skin permeability, oily texture of local carriers, and gastrointestinal disorders. Regarding systemic treatment, methotrexate (MTX) remains the first choice for cost-effectiveness and long-term record in China [13]. However, MTX is accompanied by side effects such as gastrointestinal discomfort, liver and kidney function damage, bone marrow suppression, and pulmonary fibrosis [14]. Hence, it is urgent to explore new and safe drugs that can counteract the symptoms of psoriasis.

Traditional Chinese Medicine (TCM) has been widely utilized for several years, and resources are rich and valuable in China. At present, more and more attention has been paid to exploring the utilization of TCM resources. Research enthusiasm for TCM has been stimulated globally, especially after the discovery of artemisinin by Youyou Tu from China [15]. Benzoylaconitine (BAC) is one of the most common TCMs widely used for its antiviral, anti-tumor, and anti-inflammatory pharmacological effects [16]. BAC is a lower toxic monoester type alkaloid considered to be the pharmacodynamic constituent of the *Aconitum* species, which shows potential anti-inflammatory effects [17,18,19,20]. However, the effects of BAC on psoriasis presently remain unknown. Thus, we investigated the role of BAC in the TNF-α/LPS-induced inflammatory response in HaCaT cells based on the imiquimod (IMQ)-induced mouse model. Moreover, the possible underlying mechanisms were explored with the purpose of developing effective TCM agents.

## 2. Results

### 2.1. BAC Restrains the Abnormal Proliferation but Not the Survival of HaCaT Cells

**Figure 1:**
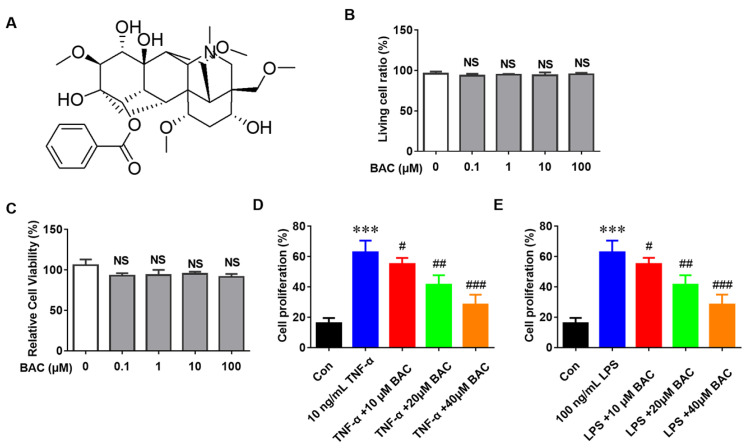
The chemical structure of BAC and its effects on cell proliferation and viability. (**A**) The chemical structure of BAC. (**B**,**C**) Cell viability was measured by Flow cytometry and CCK-8. (**D**) Detection of cell proliferation after treatment with TNF-α (10 ng/mL) and BAC (0, 10, 20, and 40 μM) for 24 h. (**E**) Detection of cell proliferation after treatment with LPS (100 ng/mL) and BAC (0, 10, 20, and 40 μM) for 24 h (Mean ± SD; vs. Con, *** *p* < 0.001; vs. TNF-α, ^#^ *p* < 0.05, ^##^ *p* < 0.01, ^###^ *p* < 0.001; NS = not significant).

Before BAC therapy, the viability of HaCaT cells was evaluated through Flow cytometry and CCK-8. The data demonstrated that cell viability was not altered after treatment with different concentrations of BAC (0, 0.1, 1, 10, and 100 μM) compared with the negative control (Con) group (Figure 1A–C). To explore the anti-proliferation effects of BAC on keratinocytes, HaCaT cells were treated with TNF-α or LPS separately. The results showed that BAC suppressed the proliferation of keratinocytes stimulated in a dose-dependent manner (Figure 1D,E), which indicated that BAC might be a potential anti-proliferation agent for keratinocytes.

### 2.2. BAC Suppressed the Production of Inflammatory Cytokines in TNF-α/LPS-Induced HaCaT Cells

**Figure 2:**
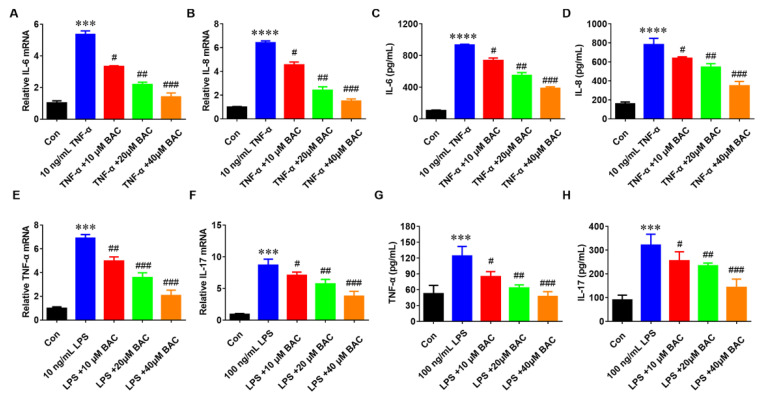
BAC significantly inhibited the gene and protein expression level of inflammatory cytokines in TNF-α/LPS-induced HaCaT cells. (**A**–**H**) mRNA level of IL-6, IL-8, TNF-α, and IL-17, and the protein level of IL-6, IL-8, TNF-α, and IL-17 in the treated cells with TNF-α (10 ng/mL) and BAC (0, 10, 20, and 40 μM), respectively. (Mean ± SD; vs. Con, **** *p* < 0.0001, *** *p* < 0.001; vs. TNF-α, ^#^ *p* < 0.05, ^##^ *p* < 0.01, ^###^ *p* < 0.001).

Massive production of pro-inflammatory cytokines is the main characteristic of psoriasis [21]. To evaluate the anti-inflammatory effect of BAC on psoriatic keratinocytes, the HaCaT cells were treated with TNF-α at different doses (0, 10, 20, and 40 μM) of BAC treatments for 24 h separately. Then, IL-6 and IL-8 expression levels in TNF-α-induced HaCaT cells were detected by qRT-PCR and ELISA. The results revealed that cytokines were significantly enhanced after treatment with TNF-α, while BAC reduced the expression levels of IL-6 and IL-8 compared with the TNF-α group (Figure 2A–D). Consistent with previous results, BAC could mitigate the LPS-induced inflammatory response. As shown in Figure 2E–H, both mRNA and protein expression of TNF-α and IL-17 were significantly reduced after treatment with BAC, which means the BAC could inhibit the release of inflammatory cytokines in LPS-stimulated HaCat cells.

### 2.3. BAC Attenuated IMQ-Induced Murine Psoriasis-like Skin Inflammation

**Figure 3:**
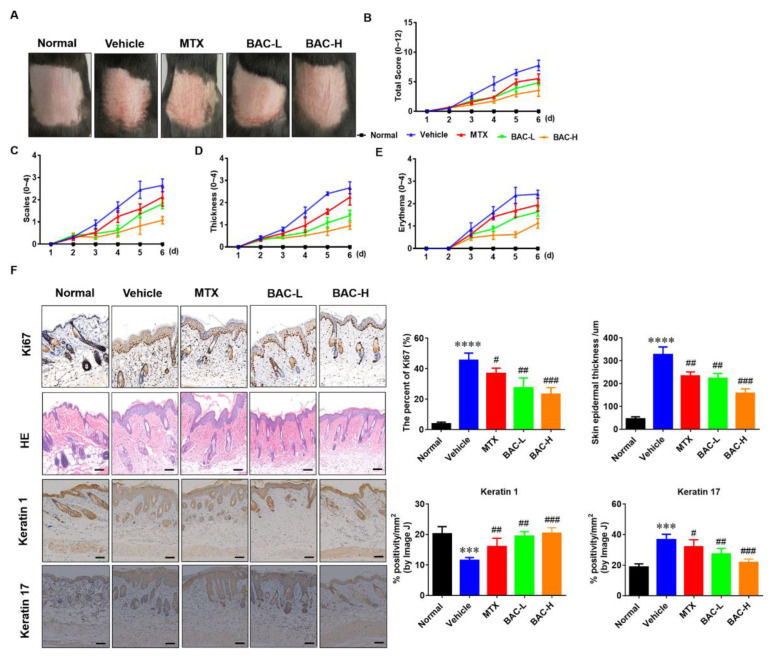
BAC attenuated IMQ-induced mouse psoriatic lesion. (**A**) The images of mouse back skin in different groups. (**B**) PASI scores of skin lesion. (**C**–**E**) The daily scales, epidermal thickness, and erythema, respectively. (**F**) H&E staining and statistics of Ki-67, Keratin 1 and Keratin 17-positive cells (Scale bar = 100 μm (200×); Mean ± SD; vs. normal, **** *p* < 0.0001,*** *p* < 0.001; vs. vehicle, ^#^ *p* < 0.05, ^##^ *p* < 0.01, ^###^ *p* < 0.001).

The accumulation of inflammatory factors, hyperproliferation of keratinocytes, and abnormal expression of parakeratosis in the skin can lead to red scaly lesions, hyperplasia, and micro-abscesses [22]. To evaluate the therapeutic effects of BAC, IMQ-induced C57BL/6 mice with psoriasis-like skin inflammation were used as the disease model. Based on this model, red scaly plaques and hyperplasia were observed in the C57BL/6 mice (Figure 3A). Therapeutically, BAC overtly attenuated the experimental symptoms in a dose-dependent manner compared with the MTX-treated group, including scales, thickness, and erythema (Figure 3B–E). The HE results showed that epidermal hyperplasia and infiltration of inflammatory cells significantly decreased the epidermal thickness and inflammatory cell infiltration in the model group after treatment with BAC. In addition, compared with the model group, the Ki-67 expression of the BAC group was significantly reduced, indicating that BAC could reverse the IMQ-induced increase of keratinocyte proliferation. BAC increased keratin 1 expression and reduced the expression of keratin 17, which contributed to an improvement in skin lesions (Figure 3F). The data indicated that BAC had an excellent therapeutic effect on psoriasis.

### 2.4. BAC Inhibited the Th17 Cells Accumulation in Model Mice

**Figure 4:**
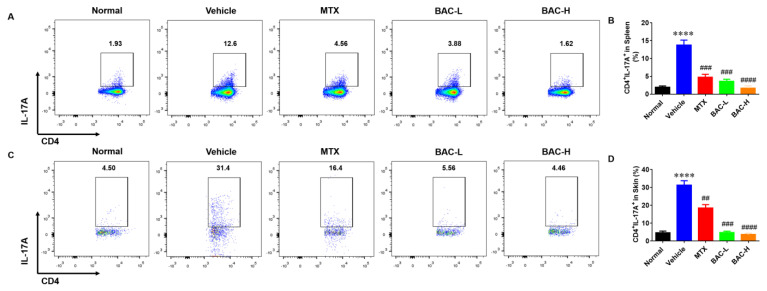
BAC reduced the accumulation of Th17 cells in IMQ-induced mice. (**A**,**B**) The frequency of Th17 cells in the spleen in IMQ-induced mice after analysis of flow cytometry. (**C**,**D**) The frequency of Th17 cells in the skin of IMQ-induced mice after analysis of flow cytometry (Mean ± SD; vs. Normal, **** *p* < 0.0001; vs. Vehicle, ^##^ *p* < 0.01, ^###^ *p* < 0.001, ^####^ *p* < 0.0001).

Previous data have attested that among immune cells, IL17A-producing Th17 helper (Th17) cells play an important role in the progression of psoriasis [23,24]. Moreover, the accumulation of Th17 cells was analyzed to verify the effects of BAC on them. The data demonstrated that a higher population of CD3^+^CD4^+^IL17A^+^Th17 cells was observed in IMQ-induced mice, while the trend was reversed after BAC treatment in the spleen and skin, which indicated that BAC inhibited the immunoreaction in the mouse model (Figure 4A–D).

### 2.5. BAC Reduced the Release of Inflammatory Cytokines in IMQ-Induced Mouse Skin

**Figure 5:**
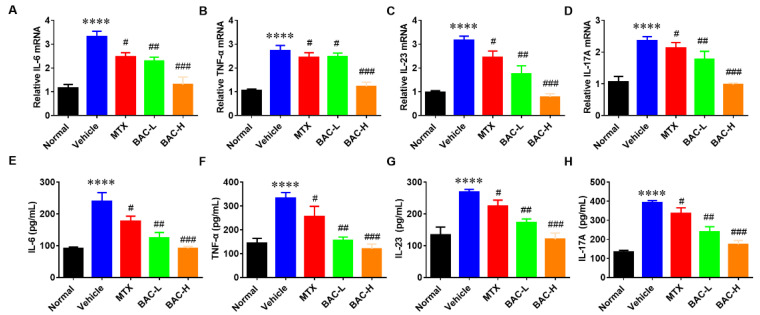
BAC significantly inhibited the gene and protein expression level of inflammatory cytokines in IMQ-induced mice. (**A**–**H**) The mRNA level of IL-6, TNF-α, IL-23, and IL-17A, and the protein level of IL-6, TNF-α, IL-23, and IL-17A, respectively (Mean ± SD; vs. Normal, **** *p* < 0.0001; vs. Vehicle, ^#^ *p* < 0.05, ^##^ *p* < 0.01, ^###^ *p* < 0.001).

Pathogenesis of psoriasis is caused by the changes in concentration and distribution of IL-12, IL-23 and IL-17. Apart from IL-12 and IL-23, IL-17, IL-6, IL-8 and interferon- γ (IFN-γ) play a vital role in the psoriatic cytokine network [24]. To deeply understand the influence of BAC on psoriasis, the release of different inflammatory cytokines was determined [25,26]. As we expected, both the gene and protein of inflammatory cytokines, including IL-6, TNF-α, IL-23, and IL-17, were increased in the skin extracts (Figure 5A–D), and the secretion of these cytokines was reduced by BAC (Figure 5E–H), which is consistent with the in vitro results.

### 2.6. Safety of BAC Administration

**Figure 6:**
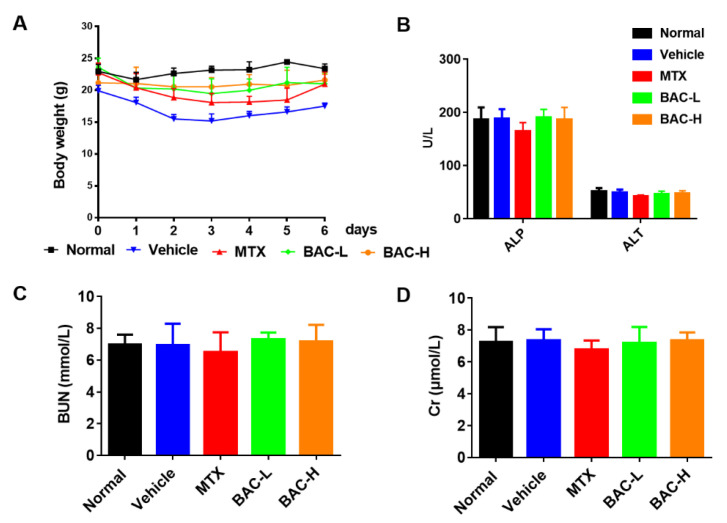
Safety analysis of BAC for dosing IMQ-induced mice. (**A**) Body weight of mice in each group. (**B**–**D**) Serum level of ALP/ALT, Cr, and Bun, respectively.

To evaluate the potential dose toxicity of BAC, the body weight of all mice was monitored daily. From the first day of feeding, the weight of the mice was significantly reduced in the IMQ group, but the phenomenon was improved with the treatment of MTX and BAC. Interestingly, it showed that mice lost less weight after treatment with BAC compared with MTX (Figure 6A). The indexes of renal and liver function indicators include serum creatinine (Cr), alkaline phosphatase (ALP), alanine aminotransferase (ALT), glutamic oxaloacetic transaminase (GOT), blood urea nitrogen (BUN), etc. [27]. Moreover, no obvious change was found in the renal and liver functions between each group (Figure 6B–D). We might conclude that dosing BAC at 2 mg/kg and 4 mg/kg is relatively safe in mice.

### 2.7. BAC-Regulated STAT3 Pathways in Keratinocytes

**Figure 7:**
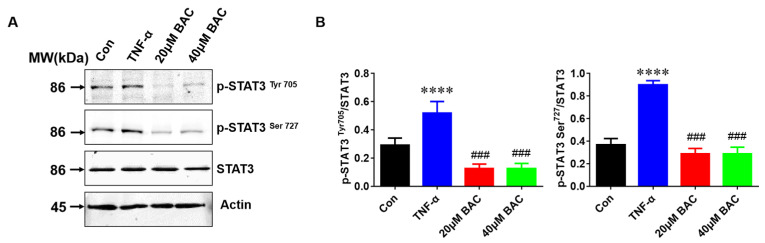
The suppression of STAT3 phosphorylation by BAC in TNF-α stimulated HaCaT cells. (**A**) The expression level of p-STAT3^Tyr 705^, p-STAT3^Ser727^, STAT3, and Actin with WB analysis in TNF-α- induced HaCaT cells. (**B**) Histogram of p-STAT3^Tyr705^, p-STAT3^Ser727^, and STAT3 expression levels in TNF-α-induced HaCaT cells (Mean ± SD; vs. Con, **** *p* < 0.0001; vs. TNF-α, ^###^ *p* < 0.001).

STAT3 distinctly contributes to the inflammatory response in psoriasis. It has been reported that sunitinib reduces imiquimod-induced psoriasis-like inflammation by inhibiting p-STAT3 [28,29]. As shown in Figure 7, the data suggested that both p-STAT3 ^Tyr705^ and p-STAT3 Tyr^727^ were significantly increased after exposure to TNF-α, especially p-STAT3 Tyr^727^. On the contrary, BAC treatment strongly inhibited STAT3 phosphorylation in TNF-α-treated HaCaT cells.

## 3. Discussion

Psoriasis is a disease that is regulated by multiple genes and inflammation. Abnormal proliferation and the differentiation of keratinocytes result in hyperplasia of the epidermis. As for patients with psoriasis, topical therapy is the conventional treatment, but its efficacy is not satisfactory owing to severe side effects, making it paramount to discover new agents [30]. TCMs, such as *Aloe vera*, *Cogon rhizome*, and *Angelica sinensis*, are attracting attention for the treatment of psoriasis [31]. BAC, one of the *Aconitum* herbs, has pharmacological anti-inflammatory effects [32]. For example, BAC inhibits the release of inflammatory cytokines in rheumatoid arthritis (RA). In the present study, we found that BAC could relieve symptoms of psoriasis through the suppression of proliferation and production of cytokines in TNF-α/LPS-induced HaCaT cells. Specifically, the proliferation rate was notably increased after stimulating with TNF-α/LPS, while BAC suppressed the proliferation and pro-differentiation in a dose-dependent manner, indicating that BAC could be a promising agent for the treatment of psoriasis.

Cytokines, including IL-6, IL-8, TNF-α, IL-23, and IL-17, are beneficial for accumulating immune cells and aggravating keratinocyte proliferation [33,34]. It has been reported that IL-6 expression could be reduced by thalidomide, leading to inhibition of the inflammatory response in psoriasis [35]. TNF-α, involved in the regulation of immune and inflammatory responses, is related to the development of psoriasis [36]. IL-23, secreted by myeloid dendritic cells (mDCs), is increased when psoriasis persists or worsens and promotes Th17 cells differentiation to produce more IL-17A and IL-17F, which are pathophysiological evidence of psoriasis [37]. The suppressive function of BAC on the inflammatory response has been identified in some disease models [38]. Given the anti-inflammatory effect of BAC, we examined the production of inflammatory cytokines in TNF-α/LPS-induced HaCaT cells accordingly. Our results indicated that BAC could reduce the expression of various inflammatory factors secreted from both TNF-α and LPS-treated HaCaT cells, benefiting from improving the inflammatory environment in skin lesions.

At present, IMQ is one of the methods widely used to establish psoriasis animal models because of its better replication of inflammatory cytokine response and immune cell infiltration in human psoriasis [39]. Therefore, we established psoriasis-like dermatitis by applying IMQ on the mice’s backs. The results clearly indicated that BAC ameliorated IMQ-induced psoriatic lesions. The PASI difference between the control group and the IMQ group narrowed after 7 days of administration of BAC. H&E staining also showed that the epidermal hyperplasia and infiltration of inflammatory cells significantly decreased the epidermal thickness and inflammatory cell infiltration in the model group after treatment with BAC. Additionally, Ki-67, keratin 1, and keratin 17 stainings were used to determine the proliferation and differentiation in IMQ-induced mice. Compared to the IMQ group, BAC reduced Ki67 and keratin 17 and promoted keratin 1 positive cells. Dependent on the IL-23/IL-17 axis, IMQ could induce inflammation in the skin and result in proinflammatory cytokine release and infiltration of immune cells. Because the T lymphocytes (especially Th17 cells) play an important role in the pathogenesis of psoriasis, our results demonstrated that BAC could reduce the infiltration of Th17 cells and reduce the serum level of IL-6, TNF-α, IL-23 and IL-17 in IMQ-induced mice, indicating that BAC could suppress inflammation on both cutaneous and systemic level.

Drug biological safety needs to be considered when developing an ideal agent to treat psoriasis [40]. For weight statistics and evaluation, our data demonstrated that body weight is not affected by BAC treatment. Renal and liver function assessment is also an important focus for a drug application. Moreover, renal and liver function did not obviously change between each group, which suggested that BAC is most likely a safe and effective drug. Studies reported that STAT3 plays a crucial role in inducing the signaling cascades of multiple cytokines and growth factors [41]. Furthermore, the STAT3 pathway was involved in the IL-6-stimulated inflammatory and catabolic phenotype of AF cells, and specifically mediates the signal transduction of many cytokines and growth factors from the cell membrane to the nucleus. This study demonstrated that BAC might act as an inhibitor of STAT3 in psoriasis to inhibit STAT3 phosphorylation in TNF-α-induced HaCaT cells to regulate the proliferation and expression of inflammatory cytokines.

In summary, our data demonstrated that BAC alleviated progression by inhibiting HaCaT cell proliferation, reducing the release of inflammatory cytokines and accumulation of Th17 cells via the STAT3 pathway to improve psoriasis. Both in vitro and in vivo studies implied that BAC could be used as a potentially effective therapeutic agent for psoriasis in the future.

## 4. Materials and Methods

### 4.1. Mice

In the present experiments, 8–10 weeks old C57BL/6 mice were purchased from Shanghai Laboratory Animal Center (Shanghai, China). Subsequently, all mice were bred and maintained under specific pathogen-free (SPF) conditions. All animal experiments in this study were approved by the Center of Animal Experiments and performed according to the institutional guidelines of Animal Ethics Certification of the Henan University of Chinese Medicine.

### 4.2. Detection of Cell Viability

HaCaT cells were purchased from Shanghai Blowing Applied Biotechnology Co., Ltd. and cultured in Dulbecco’s modified Eagle’s medium supplemented with 10% fetal bovine serum and 1% penicillin and streptomycin (Sigma-Aldrich; Merck KGaA, Darmstadt, Germany), and then incubated at 37 °C in a humidified incubator containing 5% CO_2_. Cell Counting Kit-8 (CCK-8; Dojindo Molecular Technologies, Inc., Kumamoto, Japan) was used to detect the viability of HaCaT cells. The main manipulation steps were as follows: HaCaT cells were seeded into a 96-well plate at a density of 8 × 10^3^ cells/well and then were treated with TNF-α (10 ng/mL), various concentrations of BAC (0–100 μM), and a combination of TNF-α (10 ng/mL) and BAC (0, 10, 20 and 40 μM) for 24 h separately. Consequently, a CCK-8 solution (10 μL) was added to each well for an additional 2 h. Finally, absorbance values at 450 nm were measured for each well using a microplate reader.

### 4.3. RNA Extraction and RT-qPCR

Total RNAs were extracted from HaCaT cells and mouse skin using Trizol reagent (Thermo Fisher Scientific, Waltham, MA, USA), and then 1000 ng RNA was subjected to cDNA synthesis using the High-Capacity cDNA Reverse Transcription Kit (Applied Biosystems, Foster City, CA, USA) according to the manufacturer’s instructions. Real-time PCR reactions were performed in an Applied Biosystems 7500 Real-Time PCR System. Reaction cycle conditions were as follows: 95 °C for 10 min of pre-denaturation conditions, 40 cycles at 95 °C for 15 s, 60 °C for 20 s, 72 °C for 30 s, and termination at 4 °C. The primers were designed via the website (https://pga.mgh.harvard.edu/primerbank/) (accessed on 10 August 2022) and listed in Supplementary Material Table S1.

### 4.4. Measurement of Inflammatory Cytokines by ELISA

HaCaT cells were treated with different doses (10, 20, and 40 μM) of BAC for 24 h, and then the supernatants were collected. Back skin extracts were prepared using Tissue Extraction Reagent I (Invitrogen, Camarillo, CA, USA). The protein levels of inflammatory factors were measured using ELISA kits from BioLegend (San Diego, CA, USA)/R&D systems (Minneapolis, Minnesota, MN, USA) as manufacturers’ protocols.

### 4.5. IMQ-Induced Psoriasis-like Skin Inflammation in Mice

The mice were treated with 62.5 mg IMQ on the shaved back skin and simultaneous oral administration of BAC or MTX for six consecutive days to establish a psoriasis model. The mice were randomly divided into five groups: (i) Con group; (ii) IMQ stimulated psoriasis model group; (iii) MTX (1 mg/kg); (iv) BAC-L (1 mg/kg); and (v) BAC-H (3 mg/kg) pre-treated psoriasis groups. The murine skin was evaluated by independent investigators with clinical severity scoring of erythema, scaling and thickening, based on the Psoriasis Area and Severity Index (PASI) scores (0, none; 1, slight; 2, moderate; 3, marked; and 4, very marked). The total clinical severity score was the sum of the three scores and served as a measure of disease severity (scale 0–12).

### 4.6. Skin Cell Preparation and Flow Cytometry

Cells isolated from skin lesions and spleens were prepared according to previous studies (Miroddi et al., 2015 [20]). Briefly, cells were first stained with the following different cell surface markers, Alexa Fluor 700 anti-CD45 mAb (BioLegend, USA), FITC anti-CD3 mAb (BioLegend, USA), or APC anti-CD4 mAb (BioLegend, USA). For intracellular staining of IL-17, cells were stimulated with phorbol 12-myristate 13-acetate/ionomycin for 4 h in the presence of GolgiPlug (BD Biosciences, Franklin Lakes, NJ, USA). Samples were analyzed using an LSRFortessa flow cytometer (BD Biosciences) and FlowJo software 10 (TreeStar, Woodburn, OR, USA).

### 4.7. Western Blotting

HaCaT cells were lysed in lysis buffer and centrifuged at 12,000 rpm for 20 min to collect the supernatants. After measuring protein concentration using the Bio-Rad protein assay kit (Bio-Rad, Hercules, CA, USA), 20 μg protein supernatants were separated by 10% SDS-PAGE and transferred to polyvinylidene difluoride (PVDF) membrane filters (Millipore, Billerica, MA, USA). The membranes were blocked by 5% non-fat skim milk for 1 h at room temperature (RT). After washing with TBST buffer, the membranes were incubated with the following primary antibodies (Cell Signaling Technology, Berkeley, CA, USA) overnight at 4 °C: anti-p-STAT3^Tyr705^ (1:1000) (^#^9145), anti-p-STAT3^Ser727^ (1:1000) (^#^49081), anti-STAT3 (1:1000) (^#^9139), and anti-Actin (1:5000) (^#^3700). After washing with TBST buffer, the membranes were incubated with secondary antibodies for 1 h at RT. The band density was evaluated with a computer-assisted image analysis system (Adobe Systems, San Jose, CA, USA).

### 4.8. Skin Histopathological Analysis

Back skin excised from the animals was fixed in 4% paraformaldehyde overnight and embedded in paraffin blocks. After sectioning, all samples were stained with hematoxylin and eosin (HE) to determine the inflammation infiltration. As for the HE dyeing, antibody rabbit anti-Ki-67 and polymer-horseradish peroxidase-labeled goat anti-rabbit IgG were used as the primary antibody and the secondary antibody (DAKO, Glostrup, Denmark), respectively. The positively stained epidermis was quantified by ImageJ software (National Institutes of Health, Bethesda, MD, USA).

### 4.9. Drug Safety Analyses

To determine the toxicity of BAC in vivo, the body weight of mice was monitored daily. The serum level of Cr, BUN, ALT/ALP, and the total and differential number of leucocytes were detected using a Roche Cobas C311.

### 4.10. Statistical Analysis

All data were analyzed with Graph Prism 6.0, and statistics were compared using the one-way ANOVA test (mean ± SD). Statistically significant difference was defined as *p* < 0.05. Each experiment consisted of at least three replicates per condition.

## 5. Conclusions

In summary, our data demonstrated that BAC alleviated progression through inhibiting HaCaT cell proliferation, reducing the release of inflammatory cytokines and accumulation of Th17 cells via STAT3 pathways to improving psoriasis. Both in vitro and in vivo studies implyed that BAC could be used as a potential effective therapeutic agent for psoriasis in future.

## Data Availability

The data presented in this study is contained within the article.

## Supplementary Materials

The following supporting information can be downloaded at: https://www.mdpi.com/article/10.3390/molecules28114473/s1, Table S1: Primer sequence.
