# Supplementary material for: Benzoylaconitine Alleviates Progression of Psoriasis via Suppressing STAT3 Phosphorylation in Keratinocytes
Source: Molecules. 2023 May 31;28(11):4473. doi: 10.3390/molecules28114473 (PMC10254579; doi:10.3390/molecules28114473)
Supplement: Supplementary file 1 [file molecules-28-04473-s001.zip › molecules-2361365-supplementary.pdf]

Table S1. Primer sequence.

Primer	Sequence (5′-3′)
hIL-6- F	TTCGGTCCAGTTGCCTTCTC
hIL-6- R	TCTTCTCCTGGGGGTACTGG
hIL-8-F	TCTGTCTGGACCCCAAGGAA
hIL-8- R	ATGAATTCTCAGCCCTCTTCAA
hIL-1β-F	AGCTACGAATCTCCGACCAC
hIL-1β- R	CGTTATCCCATGTGTCGAAGAA
hActin-F	GTACGCCAACACAGTGCTG
hActin-R	CGTCATACTCCTGCTTGCTG
mIL-6- F	CTGCAAGAGACTTCCATCCAG
mIL-6- R	AGTGGTATAGACAGGTCTGTTGG
mTNF-α-F	CCTGTAGCCCACGTCGTAG
mTNF-α- R	GGGAGTAGACAAGGTACAACCC
mIL-23-F	AATAATGTGCCCCGTATCCAGT
mIL-23- R	GCTCCCCTTTGAAGATGTCAG
mIL-17A-F	TTTAACTCCCTTGGCGCAAAA
mIL-17A- R	CTTTCCCTCCGCATTGACAC
mActin-F	GGCACCACTGAACCCTAAGG
mActin-R	ACAATACCAGTTGTACGTCCAGA
